# Coinfection of pulmonary tuberculosis and mucormycosis in a patient with poor controlled diabetes mellitus: A case report

**DOI:** 10.1016/j.radcr.2023.08.091

**Published:** 2023-09-18

**Authors:** Mehran shahanikelaki, Mohammad Mohammadi, Aynaz Mohammadi, Vahan Moradians

**Affiliations:** a Department of Internal Medicine, School of Medicine, Hazrat-e Rasool General Hospital, Iran University of Medical Sciences, Tehran, Iran; b School of Medicine, Iran University of Medical Sciences, Tehran, Iran; c Student Research Committee, Iran University of Medical Sciences, Tehran, Iran

**Keywords:** Mucormycosis, Tuberculosis, Coinfection, Diabetes mellitus

## Abstract

Coinfection of pulmonary mucormycosis and tuberculosis is a rare and challenging condition, particularly in immunocompromised patients. We present the case of a 60-year-old woman with poorly controlled diabetes mellitus who developed dysphonia, persistent cough, and expectoration. Imaging studies revealed a cavitary lesion and a mass in the left lung, along with stenosis of the left main bronchus. A bronchoscopy confirmed the presence of a bronchomediastinal fistula with pus discharge. Polymerase chain reaction testing of bronchial secretions revealed a co-infection of tuberculosis and mucormycosis. The patient was initiated on appropriate treatment for both infections, and her symptoms improved without disease progression. Concomitant pulmonary mucormycosis and tuberculosis pose significant diagnostic challenges due to overlapping clinical and radiological features. Early recognition and a multidisciplinary approach involving infectious disease specialists, pulmonologists, radiologists, and surgeons are crucial for optimal management. The prognosis of this coinfection is poor, emphasizing the importance of timely diagnosis and treatment. To improve outcomes, comprehensive screening and early detection of coinfections in high-risk patients, such as those with uncontrolled diabetes, are essential. Future advancements in diagnostic tools may facilitate prompt and accurate diagnosis. Clinicians should maintain a high index of suspicion and employ appropriate diagnostic techniques to ensure early identification and effective management of these complex infections.

## Introduction

Mucormycosis, a group of diseases caused by angiotrophic fungi in the order Mucorales, represents a life-threatening medical emergency and is most commonly found in immunocompromised patients [1]. The prevalence of mucormycoses among hospital discharges is estimated to be 0.12 per 10,000 [2], with rhinocerebral, pulmonary, cutaneous, gastrointestinal, disseminated, and uncommon presentations such as endocarditis, mediastinitis, peritonitis, osteomyelitis, and renal abscesses being the primary anatomical localizations [1]. A wide range of conditions compromising the immune system are identified as primary risk factors for mucormycosis. Hematologic neoplasms, neutropenia, uncontrolled diabetes mellitus, body trauma and wound contamination, glucocorticoid use, intravenous drugs, iron overload, and extreme malnutrition are among the identified risk factors [3,4].

In addition to mucormycosis, immunocompromised patients are also at high risk of developing tuberculosis, with an estimated 1.7 billion people having latent Mycobacterium tuberculosis infections worldwide [5]. Pulmonary tuberculosis, the most common form of the disease, manifests as thoracic pain, cough, fever, weight loss, hemoptysis, and dyspnea [6,7]. Diagnosis of pulmonary TB is based on clinical suspicion, clinical findings, imaging studies, and analysis of tissue and secretions.

The co-occurrence of these 2 diseases in a single patient, however, is rare. A case report documented a diabetic patient with co-infection of pulmonary tuberculosis and mucormycosis, which is a diagnostic challenge for treating physicians given their similar clinical and radiological features [8]. In another case, a young diabetic man with pulmonary mucormycosis was presented, including bronchial necrosis causing a bronchomediastinal fistula and extensive mediastinal and subcutaneous emphysema [9]. The diagnosis of mucormycosis is critical as prompt surgical control and swift medical management can significantly reduce mortality and organ loss [10]. Diagnosis of the disease requires a pathological examination of the tissue involved, as swabs and washing specimens are inadequate.

## Case presentation

A 60-year-old woman, with a complex medical history that includes poorly controlled diabetes mellitus, a past breast cancer diagnosis, and a heavy smoking habit. She presented to our center with dysphonia and a persistent cough with expectoration that had persisted for over a month, despite receiving antibiotics at another facility. Upon examination, the patient exhibited tachypnea with a respiratory rate of 26/min, was febrile, and exhibited decreased breath sounds over her left hemithorax upon chest auscultation.

The relevant Routine hematology lab tests are as follows with their reference range in parentheses: Leukocyte count = 15000 µL (4000-10,000), Hemoglobin= 11.3 g/dL (12-16), Mean corpuscular volume= 80.3 FL (80-100), Platelet count = 501,000 µL (150,000-450,000).

The relevant blood chemistry lab tests are as follows with their reference range in parentheses: Creatinine = 1.6 mg/dL (0.6-1.3), Urea nitrogen = 69 mg/dL (18-55), Ferritin= 2360 µg/L (20-110), HbA1C= 15.5% (under 5.6%), ESR 1 hour = 60 mm/h (up to 22), CRP = 115.3 mg/dL (under 6). Liver function enzymes were normal.

The venous blood gas analysis results are as follows with their reference range in parentheses: PH = 7.42 (7.32-7.43), PCO2 = 28 (38-50), bicarbonate= 18.2 mmol/L (22-26). In the hormone analysis the Galactomanan test for Aspergillus was nonreactive (0.12 with a reference range of <0.5) and also procalcitonin (ECL) was 0.139 ng/mL which is nonreactive.

**Fig. 1:**
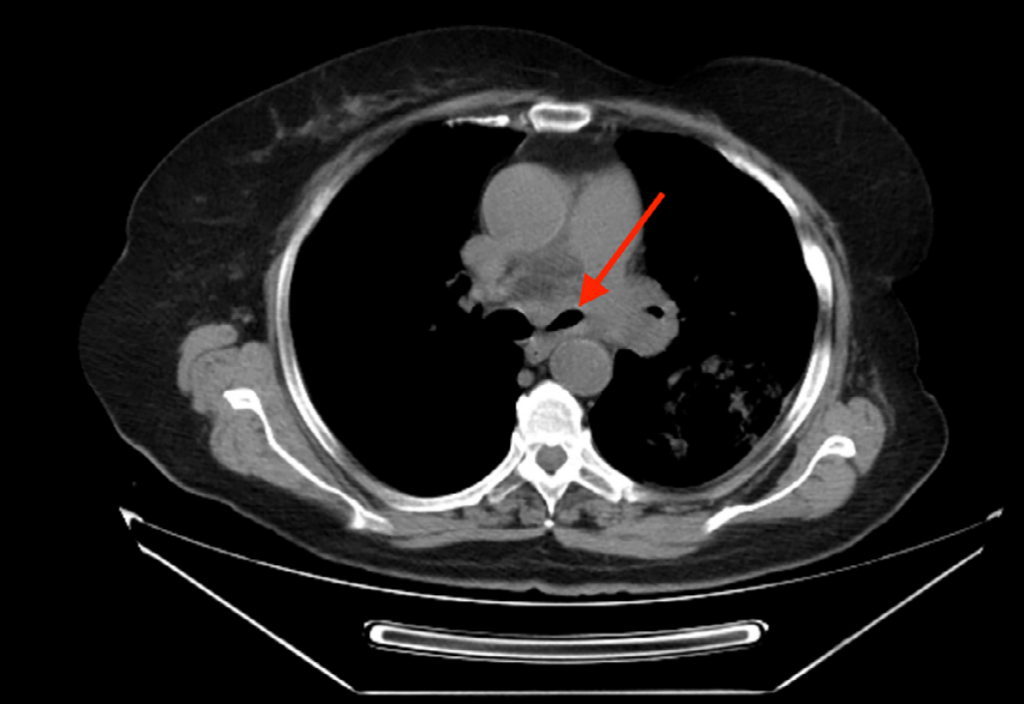
Mediastinal view; the arrow shows narrowing of the left main bronchus with suspected endobronchial lesions and infiltration in the surrounding area of the left lower bronchial lobe.

**Fig. 2:**
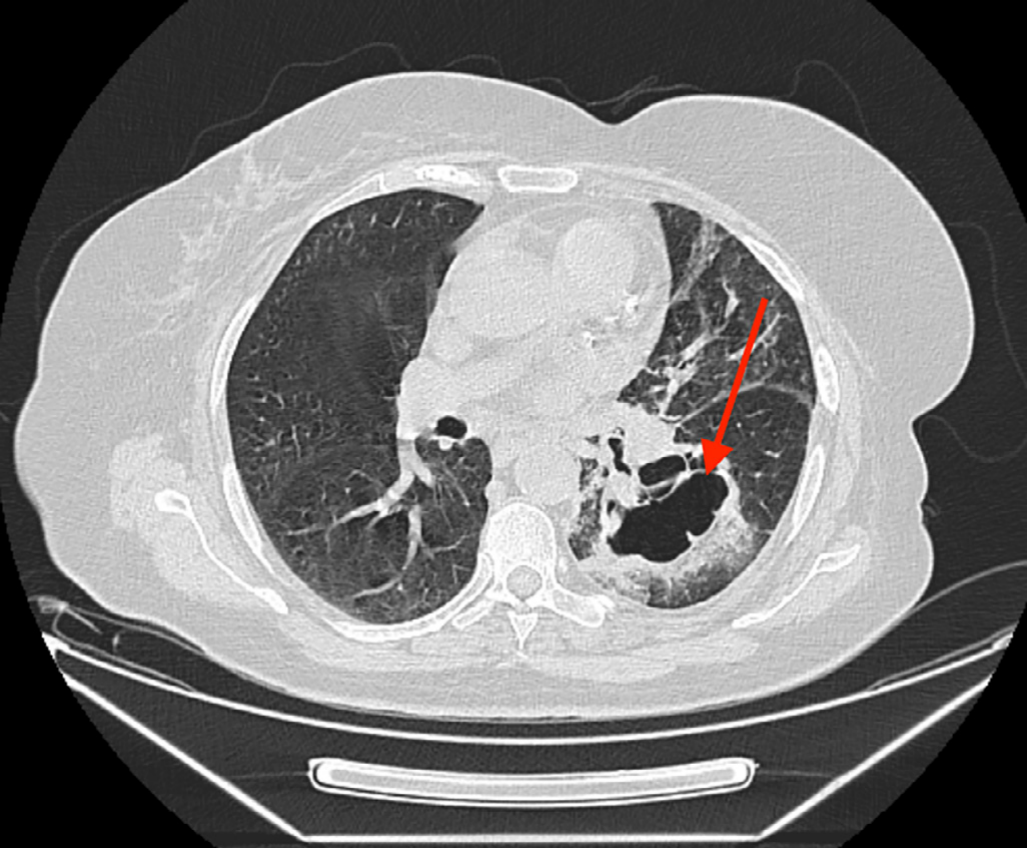
Parenchymal view; the arrow shows cavity in the upper segment of the left lower lobe with ground-glass infiltration around the cavity and in the lingula.

**Fig. 3:**
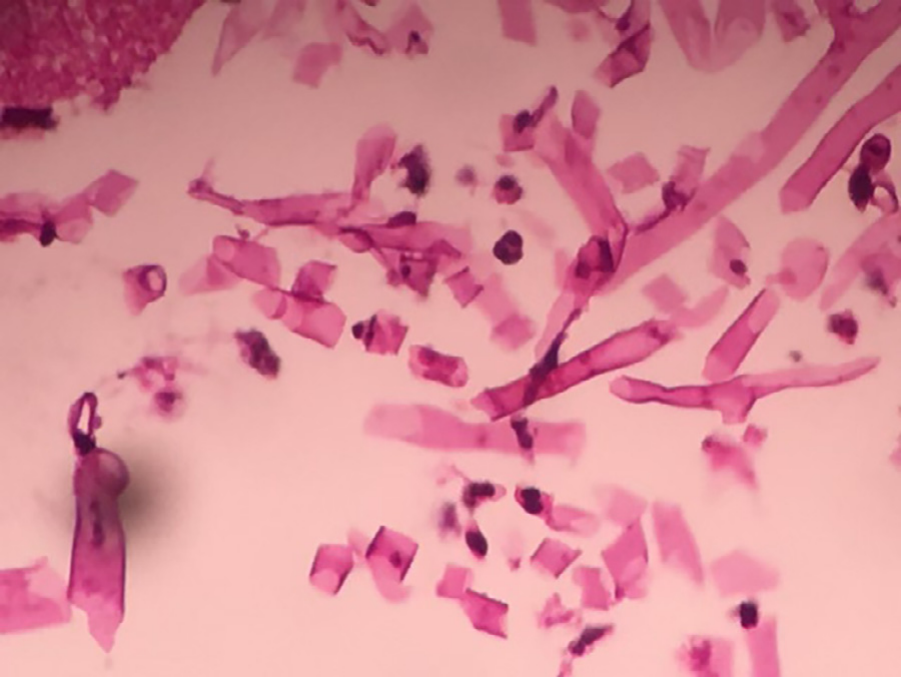
Histopathological examination of bronchial mucosa with Hematoxylin and Eosin staining and x100 magnification; showing abundant, thick, and pale hyphae without septa.

A thoracic CT scan revealed a cavitary lesion and a mass in the hilum of the left lung, as well as stenosis of the left main bronchus. (Figs. 1 and 2) The sputum culture was performed and it showed no pathogenic bacteria. The polymerase chain reaction (PCR) test of sputum was negative for BK (Koch's Bacillus), mucormycosis, and *Candida albikans*. A subsequent bronchoscopy revealed the presence of a bronchomediastinal fistula with pus discharge and minimal inflammation. PCR testing on bronchial secretions, obtained on brochoscopy, revealed a co-infection of both tuberculosis and mucormycosis. A histopathological examination of bronchial mucosa revealed abundant, thick, and pale hyphae without septa, which was consistent with a diagnosis of pulmonary mucormycosis. (Fig. 3) The patient received appropriate treatment with anti-TB therapy and liposomal amphotericin B for mucormycosis. Currently, her symptoms have improved, and the disease has not progressed further.

## Discussion

Concomitant pulmonary mucormycosis and tuberculosis is a rare but serious condition that poses significant diagnostic and management challenges. The combination of 2 opportunistic infections in a host who is immunosuppressed due to poorly controlled diabetes can lead to a range of clinical presentations, including prolonged fever, cough, hemoptysis, and chest pain, which can mimic other respiratory diseases such as pneumonia, aspergillosis, tuberculosis or tumoral infiltration [4]. Moreover, the radiographic findings of pulmonary mucormycosis, such as parenchymal infiltrates, nodules, cavitation, and effusion, are similar to those of tuberculosis, further complicating the diagnosis [11].

The reported incidence of concomitant pulmonary mucormycosis and tuberculosis is low, with only a few cases reported in the literature [8,[12], [13], [14]] In the case presented above, an immunosuppressed patient due to uncontrolled diabetes mellitus developed pulmonary mucormycosis and tuberculosis simultaneously, leading to respiratory failure and a prolonged hospital stay. The patient was started on antifungal therapy due to the fatality of untreated mucormycosis. The coinfection was managed with a combination of antifungal and antitubercular drugs and the outcome was good.

The diagnosis of concomitant pulmonary mucormycosis and tuberculosis is challenging due to the nonspecific clinical and radiographic findings and the low diagnostic yield of sputum or bronchoalveolar lavage fluid cultures [10]. Invasive tissue sampling is often required to confirm the diagnosis, but it is associated with significant risks and complications in immunosuppressed patients [15]. As a result, the diagnosis is often delayed, leading to worse outcomes and increased mortality rates.

The management of concomitant pulmonary Mucormycosis and tuberculosis requires a multidisciplinary approach, involving infectious disease specialists, pulmonologists, radiologists, and surgeons. The choice of antifungal and antitubercular drugs, the duration of therapy, and the need for surgical intervention depending on the severity of the disease, the extent of tissue involvement, and the host factors [16]. Conventional or liposomal amphotericin B is the recommended antifungal therapy for mucormycosis [3], while a combination of isoniazid, rifampicin, pyrazinamide, and ethambutol is the standard antitubercular regimen. However, the concomitant use of these drugs can lead to drug interactions, adverse effects, and noncompliance, further complicating the management.

The prognosis of concomitant pulmonary mucormycosis and tuberculosis is poor, with mortality rates as high as 70% in invasive pulmonary mucormycosis [17]. The likelihood of survival depends on various factors, including the underlying host immune status, the severity and extent of tissue involvement, the timely diagnosis and treatment, and the presence of other comorbidities.

The rising burden of mucormycosis in immunosuppressed patients, particularly those with uncontrolled diabetes mellitus, highlights the need for comprehensive screening and early diagnosis of coinfections with tuberculosis or other respiratory pathogens. A high index of suspicion, careful clinical evaluation, and judicious use of imaging and laboratory tests can aid in the prompt recognition and management of these complex infections. Furthermore, the development of new diagnostic tools, such as PCR and antigen detection assays, may improve the accuracy and speed of diagnosis in the future.

## Conclusion

In high-risk patients for pulmonary fungal infections, particularly those presenting with cavitation on chest radiographs and negative sputum smear for AFB, pulmonary mucormycosis should be considered. This is especially important in countries where the prevalence of tuberculosis is high. Diagnosis of fungal pneumonia typically requires the identification of fungi within the pulmonary parenchyma via lung biopsy. However, in cases where lung biopsy is contraindicated, bronchoalveolar lavage may serve as a useful and safe diagnostic tool for the detection of mucormycosis.

## Patient consent

We hereby confirm that written, informed consent has been obtained from the patient, for the publication of her case and related information. The purpose of obtaining this consent is to ensure the patient's involvement in contributing valuable medical knowledge and to promote advancements in healthcare practices. It is understood that all personal identifying information will be kept confidential, and the case will be presented in an anonymous manner.
